# Multiplex PCR test as an intra-operative diagnostic tool for periprosthetic joint infection in presumed aseptic revision hip and knee arthroplasty: a 1-year follow-up study of 200 cases

**DOI:** 10.5194/jbji-9-9-2024

**Published:** 2024-01-24

**Authors:** Thomas J. A. van Schaik, Petra J. C. Heesterbeek, Job L. C. van Susante, Wim H. C. Rijnen, Jon H. M. Goosen

**Affiliations:** 1 Department of Orthopaedic Surgery, Sint Maartenskliniek, Nijmegen, the Netherlands; 2 Department of Orthopaedic Surgery, Rijnstate Ziekenhuis, Arnhem, the Netherlands; 3 Department of Orthopaedic Surgery, Radboud University Medical Center, Nijmegen, the Netherlands; 4 Department of Research, Sint Maartenskliniek, Nijmegen, the Netherlands

## Abstract

Automated custom-made multiplex PCR techniques (mPCR) have become commercially available and are designed for intra-operative screening of concurrent periprosthetic joint infections (PJIs). The purpose of this study was to evaluate the value of a positive mPCR test in presumed aseptic revision total hip (THA) and knee (TKA) arthroplasties after a 1-year follow-up. In an earlier study, such an automated mPCR technique (Unyvero ITI G2; Curetis, Holzgerlingen, Germany) was tested on intra-operatively obtained synovial fluid in 200 patients with a presumed aseptic TKA or THA revision. At the time of revision, no therapeutic consequences were attached to a positive test result since treating personnel were blinded for the test results. We retrospectively reviewed the outcome of cases with respect to the occurrence of PJIs using the European Bone and Joint Infection Society (EBJIS) criteria during a 1-year follow-up postoperatively. A total of 10 out of 200 patients had a positive mPCR test result at the time of revision. Of these 10 cases, none encountered outcome parameters fulfilling the criteria to diagnose PJIs in the first year after surgery, and one required re-revision surgery for reasons other than infection. Of the other 190 negative mPCR cases, none developed a PJI. A positive mPCR test at the time of presumed aseptic revision surgery did not correspond with intra-operatively obtained tissue cultures, and none of the encountered positive mPCR tests had developed a PJI at the 1-year follow-up. We recommend careful evaluation and monitoring of modern diagnostic tests before widespread use.

## 1 Introduction

Diagnosing periprosthetic joint infection (PJI) remains a challenge for orthopaedic surgeons and has a major impact on patients and healthcare systems (Middleton et al., 2019; Kurtz et al., 2012). The diagnosis of PJI is based on clinical findings, laboratory tests of peripheral blood and synovial fluid, histological evaluation of periprosthetic tissue, and intra-operative findings (Parvizi et al., 2018; McNally et al., 2021). Culture results of intra-operatively collected tissue samples may take up to 14 d to obtain, and the reported sensitivity is limited, ranging from 39 to 70 % (Corvec et al., 2012; Moran et al., 2010; Peel et al., 2016; Tande and Patel, 2014). In addition, the prevalence of unexpected positive intra-operative cultures (UPICs) in presumed aseptic revision hip and knee arthroplasty is estimated at around 10 % and results in a higher re-revision rate (Jacobs et al., 2017; Purudappa et al., 2020; Kloos et al., 2022). Therefore, the search for accurate and faster diagnostic methods remains relevant, and numerous novel methods have been proposed over the years. Specifically, the use of molecular techniques is quickly spreading in the field of PJI diagnostics, including methods based on electrospray ionisation (ESI-TOF MS) and matrix-assisted laser desorption ionisation time-of-flight mass spectrometry (MALDI-TOF MS), Fourier transform near-infrared spectroscopy (FT-NIRS), next-generation sequencing (NGS), and polymerase chain reaction (PCR) (Jacovides et al., 2012; Harris et al., 2010; Tidwell et al., 2015; Rak et al., 2016; Swearingen et al., 2016).

In a previous study, the diagnostic accuracy of the automated multiplex polymerase chain reaction (mPCR) Unyvero Implant and Tissue Infection G2 cartridge (U-ITI G2 system; Curetis, Holzgerlingen, Germany) was evaluated on intra-operatively obtained synovial fluid by comparison with the outcome of six periprosthetic tissue cultures, also obtained intra-operatively in presumed aseptic total knee and hip revisions (Jacobs et al., 2021). The specificity (Spe) and negative predictive value (NPV) were found to be high in both the knee revision group (Spe of 96.8 %, NPV of 96.8 %) and the hip revision group (Spe of 96.6 %, NPV of 92.5 %), with 16 mismatches occurring between the mPCR test result and tissue cultures (Jacobs et al., 2021).

The purpose of this cohort study of 200 patients was to evaluate the value of a positive automated mPCR test of intra-operatively obtained synovial fluid with respect to the occurrence of a PJI during the first year after a revision procedure.

## 2 Methods

We retrospectively reviewed electronic health records of all 200 patients where mPCR was performed on the intra-operatively obtained synovial fluid which was collected during revision surgery of their total knee (TKA) or total hip arthroplasty (THA) between March 2018 and November 2018 at our institution (Jacobs et al., 2021). No patients were treated with antibiotics prior to the revision procedure; however, all patients received 2 g of cefazolin as antibiotic prophylaxis at least 30 min prior to the surgery. During revision surgery, six periprosthetic tissue samples were routinely collected using sterile surgical equipment. According to our institutional protocol, antibiotic therapy (1000 mg of cefazolin, three times a day) was continued until the preliminary results of the tissue cultures were available. All treating physicians were blinded for the mPCR results, and the results therefore did not influence any therapeutic decisions.

We collected outcomes from the electronic health record regarding the development of infectious events, with or without surgery, during the first year of follow-up. The validation and application of the used mPCR technique has been described in our previous report (Jacobs et al., 2021). Local institutional review board approval (decision 1048) was obtained, and, due to the retrospective nature of this study, the consent requirement was waived. To determine the presence of PJI within 1 year after index surgery, the following outcomes were collected from the electronic health records and added to the study database, which already contained age, sex, and body mass index (BMI): microbiological results of tissue cultures, basic serology results (leucocyte count and C-reactive protein), and antibiotic administration. We have used the European Bone and Joint Infection Society (EBJIS) definition for the diagnosis of PJI (McNally et al., 2021). Despite being included in the EBJIS definition, histology is not routinely performed at our institution for the diagnostic work-up of PJI. Re-operations after the index revision procedure, such as debridement antibiotics and implant retention (DAIR) and re-revision surgery, were also recorded.

We have formed groups based on the mPCR result and the tissue culture result of the initial revision surgery (Table 1). Based on the EBJIS criteria, the diagnosis of PJI was made when at least two out of six positive tissue cultures with the same micro-organism were found. This resulted in the following four groups: (1) positive mPCR and ≥ 2 positive intra-operative tissue cultures, (2) positive mPCR and < 2 positive intra-operative tissue cultures, (3) negative mPCR and ≥ 2 positive intra-operative tissue cultures, and (4) negative mPCR and < 2 positive intra-operative tissue cultures. The occurrence rate of PJI was expressed as a percentage using descriptive statistics, performed using SPSS 28.0 (SPSS Inc., Chicago, IL).

**Table 1:** Defined groups based on mPCR test and intra-operative tissue culture of initial presumed aseptic revision surgery.

	≥ 2 positive	< 2 positive	Total
	tissue cultures	tissue cultures	
Positive mPCR	3	7	10
Negative mPCR	12	178^*^	190
Total	15	185	200

mPCR multiplex polymerase chain reaction. ^*^ This includes four invalid mPCR test results.

**Table 2:** The 1-year follow-up of cases with a positive mPCR test result ( n=10 ).

Case	Joint	mPCR synovial fluid	Tissue cultures	Antibiotics + duration	Re-revision surgery	Tissue cultures
9	Hip	CNS	*Staphylococcus saccharolyticus*	Clindamycin, 12 weeks	–	–
40	Hip	CNS	–	–	–	–
41	Knee	CNS	–	–	–	–
47	Knee	CNS	–	–	–	–
73	Knee	CNS	–	–	–	–
95	Hip	*Acinetobacter baumannii*	–	–	–	–
101	Hip	*Cutibacterium acnes*	*C. acnes* *Staphylococcus warneri*	Clindamycin, 12 weeks	Femoral stemrevision D/T stem subsidence	No growth
133	Knee	CNS	–	–	–	–
151	Hip	CNS	–	–	–	–
264	Hip	*C. acnes*	*C. acnes*	Clindamycin, 12 weeks	–	–

CNS indicates coagulase negative staphylococci; D/T indicates due to; DAIR indicates debridement, antibiotics, and implant retention; and mPCR indicates multiplex polymerase chain reaction.

**Table 3:** The 1-year follow-up of cases with positive tissue cultures and a negative mPCR result ( n=12 ).

Case	Joint	Tissue cultures	Antibiotics + duration	Two-stagerevision	Re-revision surgery	Tissue cultures
029	Knee	*Cutibacterium acnes*	Clindamycin, 12 weeks	–	–	–
058^*^	Hip	Anaerobic cocci	Clindamycin, 6 weeks	Yes	–	–
085	Hip	*C. acnes*	Clindamycin, 12 weeks	–	–	–
090	Knee	*C. acnes*	Amoxicillin, 12 weeks	–	–	–
145	Hip	*Staphylococcus lugdunensis*	Clindamycin/rifampicin, 12 weeks	–	–	–
150^*^	Hip	*Staphylococcus epidermidis*	None	Yes	ORIF D/Tperiprosthetic femoral fracture	No growth
168	Hip	*C. acnes*	Clindamycin, 12 weeks	–	–	–
171	Hip	*Staphylococcus saccharolyticus*	Clindamycin/rifampicin, 12 weeks	–	–	–
182	Hip	*S. epidermidis*	Clindamycin/rifampicin, 12 weeks	–	–	–
219	Hip	*C. acnes*	Clindamycin, 12 weeks	–	–	–
238	Knee	*S. epidermidis*	Clindamycin/rifampicin, 12 weeks	–	–	–
274	Hip	*C. acnes*	None	–	–	–

D/T indicates due to, mPCR indicates multiplex polymerase chain reaction, ORIF indicates open reduction and internal fixation, and PJI indicates periprosthetic joint infection. ^*^ These two patients underwent two-stage revision.

## 3 Results

None of the 200 patients developed an infectious event within 1 year after revision surgery. One patient had passed away within 1 year after revision surgery for reasons unrelated to the arthroplasty surgery. At the time of the index revision surgery, a total of 10 patients had a positive mPCR result (THA: n=6 ; TKA: n=4 ) (Table 2), and four patients had an invalid mPCR result. A total of 12 patients had two or more positive intra-operative cultures with a negative mPCR result (Table 3).

### 3.1 Positive mPCR and ≥ 2 positive intra-operative tissue cultures ( n=3 )

In three cases (1.5 %), the mPCR results and at least two tissue cultures were positive. The mPCR and culture results were concordant in all three cases: *Cutibacterium acnes* ( 2× ) and coagulase-negative *staphylococci* ( 1× ). In all three cases, patients were treated with a 3-month regimen of oral antibiotics. None of these cases developed an infectious event within 1 year after revision surgery. One patient underwent aseptic femoral stem revision due to stem subsidence with negative intra-operative tissue cultures.

### 3.2 Positive mPCR and < 2 positive intra-operative tissue cultures ( n=7 )

In seven cases (3.5 %), the mPCR results were positive, with tissue cultures being negative. None of these cases was treated with antibiotics, and none developed an infectious event or required revision surgery within 1 year after revision surgery.

### 3.3 Negative mPCR and ≥ 2 positive intra-operative tissue cultures ( n=12 )

In 12 cases (6 %), the mPCR results were negative, but the tissue cultures showed growth of at least two of the same micro-organisms. Nine cases (75.0 %) were treated with a 3-month regimen of oral antibiotics, and one case (8.3 %) did not receive any antibiotic treatment since the treating surgeon did not consider a PJI to be likely. None of these 10 cases developed an infectious event or required revision surgery within 1 year after revision surgery. However, 2 of the 12 cases (16.7 %) were found to be highly suspicious with regard to an infection intra-operatively (pus and/or suspected infectious tissue) despite a thorough diagnostic work-up before the revision procedure to rule out PJI.

- In the first case, only extraction took place, and an antibiotic spacer was implanted. Cultures turned out to be positive, and a 6-week oral antibiotic regimen was admitted before re-implantation as part of a two-stage procedure with negative tissue cultures after re-implantation. No infectious event occurred, and no revision surgery was performed within 1 year after the two-stage revision surgery.
- In the second case, the prosthesis was not removed, and only intra-operative cultures were obtained, which turned out to be positive. Within 1 month after initial surgery, a two-stage revision surgery was performed with an 8-week antibiotic period in between the two stages. The tissue cultures after re-implantation showed no growth. The patient was treated with a 6-week oral antibiotic regimen. No infectious event occurred within 1 year after the two-stage revision surgery.

### 3.4 Negative mPCR and < 2 positive intra-operative tissue cultures ( n=174 )

In 174 cases (87.0 %), both the mPCR and the tissue cultures were negative. In three cases with persistent wound drainage, a DAIR after THA was performed with negative tissue cultures. None of these 174 cases developed an infectious event, and surgery with an aseptic aetiology was performed in four cases with a THA and in five cases with a TKA within 1 year after initial revision surgery.

### 3.5 Invalid mPCR results ( n=4 )

In four cases (2.0 %), the mPCR results were invalid, and subsequent tissue cultures showed no growth. None of these cases developed an infectious event or required revision surgery.

**Table 4:** Review and comparison of available literature on mPCR Unyvero ITI kit.

Study	mPCR U-ITI kitused (G1/G2)	Population	Type of sample	Total patients	Total PJI (%)	Joint	SN, %	SP, %	PPV, %	NPV, %
Borde et al. (2015)	G1	Septic and aseptic revisions	Tissue	54	10 (19)	Hip, knee	NR	NR	NR	NR
Hischebeth et al. (2016)	G1	Septic and aseptic revisions	Sonicate and synovial fluid	31	18 (58)	Hip, knee, shoulder	66.7	100.0	100.0	68.4
Prieto-Borja et al. (2017)	G1	Septic and aseptic revisions	Sonicate fluid	68	29 (43)	Hip, knee, shoulder	60.5	98.0	95.8	76.6
Villa et al. (2017)	G1	Early and late PJI	DTT eluate and synovial fluid	47	47 (100)	Hip, knee, shoulder	34.2	NR	NR	NR
Malandain et al. (2018)	G1	Septic and aseptic revisions	Synovial fluid and tissue	239	NR	Hip, knee, shoulder, elbow	NR	NR	NR	NR
Lausmann et al. (2017)	G1	Septic and aseptic revisions	Synovial fluid	60	34 (57)	Hip, knee	78.8	100.0	100.0	79.4
Sigmund et al. (2019)	G1	Septic and aseptic revisions	Synovial fluid	90	38 (42)	Hip, knee, shoulder, elbow, ankle	71.1	96.2	93.1	82.0
Aamot et al. (2019)	G1	Acute PJI	Tissue	15	15 (100)	NR	73.0	NR	NR	NR
Suren et al. (2020)	G2	Septic and aseptic revisions	Synovial fluid	26	15 (58)	Hip, knee	80.0	100.0	100.0	77.0
Lausmann et al. (2020)	G2	Septic and aseptic revisions	Synovial fluid	97	47 (48)	Hip, knee	85.1	98.0	97.6	87.5
Lafeuille et al. (2021)	G2	Septic and aseptic revisions	Synovial fluid and tissue	40^a^	35 (88)^a^	Hip, knee,humerus, ankle/foot, spine	72.1	98.9	83.8	97.8
Zannoli et al. (2021)	G1	Septic and aseptic revisions	DTT eluate and sonicate fluid	43	NR	NR	NR	NR	NR	NR
Jacobs et al. (2021)	G2	Presumed aseptic revisions	Synovial fluid	200	14 (7)	Hip, knee	36.4^b^/96.8^c^	96.6^b^/96.8^c^	57.1^b^	92.5^b^
Lüdemann et al. (2022)	G1	Septic and aseptic revisions	Synovial fluid	50	14 (28)	Hip, knee, shoulder	33.0	91.0	57.0	NR
Auñón et al. (2022)	G1	Suspected PJI	Sonicate and synovial fluid, and tissue	99	99 (100)	Hip, knee, shoulder, elbow	NR	NR	NR	NR

DTT indicates Dithiothreitol, G1 indicates first generation, G2 indicates second generation, mPCR U-ITI indicates multiplex polymerase chain reaction Unyvero i60 Implant and Tissue Infection, NPV indicates negative predictive value, NR indicates not reported, PJI indicates periprosthetic joint infection, PPV indicates positive predictive value, SN indicates sensitivity, and SP indicates specificity. ^a^ Among the 40 patients included, 28 had foreign material and 12 had no foreign material before surgery. Among the 35 patients that had an infection, 23 had PJI, 6 had osteitis, 4 had soft tissue infections (STIs), 1 had spondylitis, and 1 had osteoarthritis, whereas 5 had no OAI; ^b^ PCR results for hip; ^c^ PCR results for knee.

## 4 Discussion

The most important finding of the present study was that the mPCR test showed no additional value in predicting underlying PJI in this series of 200 presumed aseptic revision cases. To our knowledge this is the first study to evaluate the value of an automated mPCR system with a follow-up period of 1 year.

The automated mPCR Unyvero system is a commercial PCR technique designed for diagnosing PJIs. Our previous study (Jacobs et al., 2021) showed a high specificity and negative predictive value (NPV) and a low sensitivity and positive predictive value (PPV) for the mPCR Unyvero ITI G2 system. Diagnostic properties of this mPCR system were analysed by numerous other studies, reporting an excellent specificity ranging from 90 % to 100 %, indicating a positive result being truly positive with high probability (Metso et al., 2014; Hischebeth et al., 2016; Morgenstern et al., 2018; Sigmund et al., 2019, 2020; Suren et al., 2020; Lüdemann et al., 2022; Auñón et al., 2022). However, the same studies reported a low sensitivity, ranging from 40 % to 80 %, meaning that a negative result does not exclude infection. In fact, orthopaedic surgeons are actually in need of a test with a high negative predictive value in order to exclude PJI in cases where an infection is unclear.

There is a difference between the previously reported diagnostic value of the mPCR test and the present results (Table 4). It is important to mention that these studies only analysed the results in the direct postoperative period and did not include a follow-up period. Some studies did not even report any diagnostic values (Borde et al., 2015; Malandain et al., 2018; Zannoli et al., 2021; Auñón et al., 2022) or only mentioned the sensitivity of the mPCR (Villa et al., 2017; Aamot et al., 2019). In addition, the type of sample being analysed was different amongst studies: four studies analysed sonicate fluid (Hischebeth et al., 2016; Prieto-Borja et al., 2017; Zannoli et al., 2021; Auñón et al., 2022), two studies analysed tissue alone (Borde et al., 2015; Aamot et al., 2019), two studies analysed dithiothreitol (DTT) eluate (Villa et al., 2017; Zannoli et al., 2021), and most studies analysed synovial fluid alone (Lausmann et al., 2017; Sigmund et al., 2019; Suren et al., 2020; Lausmann et al., 2020; Jacobs et al., 2021; Lüdemann et al., 2022) or in combination with other aforementioned samples (Hischebeth et al., 2016; Villa et al., 2017; Malandain et al., 2018; Auñón et al., 2022; Lafeuille et al., 2021). Furthermore, there was a difference in the type of joint aspirated in each study, whereas in almost every case the hip and knee joint were aspirated, and occasionally, the shoulder, elbow, ankle, or spine were aspirated.

Studies reporting on the commercial multiplex PCR kits had a relatively high prevalence of PJI in their study population, ranging from 42 % to 75 % (Metso et al., 2014; Hischebeth et al., 2016; Morgenstern et al., 2018; Sigmund et al., 2019; Suren et al., 2020). The previous study by Jacobs et al. (2021) is the only study that focused on the exclusion of PJI by PCR in patients with presumed aseptic aetiology based on other criteria and regular serology and synovial tests. In contrast, all other studies included both septic and aseptic revisions, resulting in very heterogeneous results, with a higher a priori chance of positive mPCR test results.

Despite a thorough work-up ruling out PJI before a revision procedure, 15 of the 200 cases turned out to have unexpected positive cultures (UPICs) with a described higher probability of developing infectious events postoperatively (Jacobs et al., 2021). In the literature, there is no consensus on the interpretation and treatment of these cases, including supervised neglect with or without oral and intravenous antibiotics or even chronic antibiotic suppression (Fernandez-Sampedro et al., 2015; Saleh et al., 2014; Ribera et al., 2014; Moojen et al., 2010; Marculescu et al., 2005). A recent systematic review (Kloos et al., 2022) on UPICs in TKA concluded that the heterogeneity in results amongst included papers hindered the authors in providing recommendations on the treatment of UPIC in TKA. So, to date, UPICs are considered to be uncomfortable findings after presumed aseptic surgery and warrant the need for better preoperative diagnostic tests in revision arthroplasties.

### 4.1 Limitations

A limitation of this study was that the collected data were retrospectively retrieved during a chart review, which theoretically may have contributed to missing PJIs. Secondly, we may have possibly missed some (late) PJIs occurring later than 1 year after index surgery because of the follow-up period of 1 year. However, from the results presented a with 1 year follow-up, no change in conclusions is anticipated from an extended follow-up period. Further, we have focused on only aseptic presumed revisions, which is a select group of cases. Our results are therefore not readily comparable to other studies with other selected populations. Finally, a possible limitation of this study is the absence of histology to evaluate the relevance of our conventional cultures since this is not common practice at our institution.

### 4.2 Conclusion

In this study a positive mPCR test at the time of presumed aseptic hip and knee arthroplasty revision did not correspond with the outcome of the concomitantly obtained tissue cultures. In addition, none of the 10 positive mPCR tests encountered developed a prosthetic joint infection at the 1 year follow-up. We recommend careful evaluation and monitoring of the clinical relevance of these modern diagnostic tests before widespread use can be recommended.

## Data Availability

The authors confirm that the data supporting the findings of this study are available within the article.
